# Systemic Inflammation and Central Neuronal Damage: The Relationship Between Neutrophil‐to‐Lymphocyte Ratio and Neuron‐Specific Enolase in Acute Ischemic Stroke

**DOI:** 10.1002/iid3.70456

**Published:** 2026-06-04

**Authors:** Ning Rong, Hongyan Li, Siyu Qian, Yiting Ya, Hongfu Tian, Ningning Wang, Yuehua Yu, De‐Sheng Zhu, Min‐Yuan Liu

**Affiliations:** ^1^ Department of Neurology The Fourth People's Hospital Affiliated to Tongji University Shanghai China; ^2^ Department of Neurology, Ren Ji Hospital Shanghai Jiao Tong University School of Medicine Shanghai China; ^3^ Department of Neurology Qingpu Branch of Zhongshan Hospital Affiliated to Fudan University Shanghai China; ^4^ Department of Neurology 411 Hospital of Shanghai University Shanghai China; ^5^ Department of Neurology, Shanghai Public Health Clinical Cente Fudan University Shanghai China

**Keywords:** acute ischemic stroke, biomarker, inflammation, neuron‐specific enolase, neutrophil‐to‐lymphocyte ratio

## Abstract

**Background and Purpose:**

The systemic inflammatory response, quantified by the neutrophil‐to‐lymphocyte ratio (NLR), and central neuronal injury, reflected by serum neuron‐specific enolase (NSE), are implicated in the pathogenesis of acute ischemic stroke (AIS). However, the association between these two processes remains inadequately explored at the population level. This study aimed to investigate the relationship between NLR and neuronal injury via circulating biomarkers in AIS.

**Methods:**

In this cross‐sectional study, 4272 consecutively enrolled AIS patients from a single center in China were included. Serum NSE levels were measured, and NLR was calculated from peripheral blood counts and log₂‐transformed for analysis. The association between log_2_(NLR) and elevated NSE (≥ 16.3 μg/L) was evaluated using multivariable logistic regression models adjusted for potential confounders, smooth curve fitting, and cumulative incidence analysis.

**Results:**

Serum NSE levels and the prevalence of high NSE both increased significantly across tertiles of log_2_‐transformed NLR (both *p* < 0.001). A linear relationship was observed between log_2_(NLR) and NSE levels. After full adjustment for covariates, each unit increase in log_2_(NLR) was associated with a 38% increased risk of high NSE (OR = 1.38, 95% CI: 1.25–1.53, *p* < 0.001). Receiver operating characteristic (ROC) analysis yielded an area under the curve (AUC) of 0.62 (95% CI: 0.60–0.64) for log_2_(NLR) in discriminating patients with high NSE.

**Conclusion:**

Elevated NLR is independently associated with higher levels of serum NSE in patients with AIS, supporting a link between systemic inflammation and the extent of central neuronal injury. These findings provide epidemiological evidence for an interaction between circulatory inflammatory responses and brain damage following ischemic stroke. However, due to the cross‐sectional design, causality cannot be inferred, and the modest discriminatory ability of NLR limits its potential as a standalone clinical marker.

## 1 Introduction

Acute ischemic stroke (AIS) continues to be a major cause of death and disability worldwide, presenting a growing challenge to healthcare systems [1, 2]. The pathological progression of AIS involves not only initial ischemic necrosis but also a pronounced inflammatory reaction that contributes to secondary brain injury [3]. Shortly after arterial occlusion, irreversible neuronal loss occurs within the ischemic core. This is followed by a rapid activation of inflammatory pathways, mediated largely by peripheral immune cells and inflammatory cytokines, which disrupt the blood–brain barrier and promote further neuronal degeneration. Such interactions underscore the dual pathological nature of AIS, in which ischemic and inflammatory mechanisms collectively amplify tissue damage [4, 5].

The identification of biomarkers capable of concurrently evaluating systemic inflammatory states and neuronal injury is of considerable clinical relevance. The neutrophil‐to‐lymphocyte ratio (NLR), a derived hematologic parameter, reflects the equilibrium between pro‐inflammatory neutrophils and immunoregulatory lymphocytes. Elevated NLR values have been repeatedly linked to adverse short‐term outcomes following AIS [6]. Similarly, neuron‐specific enolase (NSE), a glycolytic enzyme abundant in neurons, enters the circulation upon neuronal damage and has been established as a reliable indicator of the severity of central nervous system injury. Higher concentrations of serum NSE are associated with increased infarct volume and poorer recovery [7].

Although both NLR and NSE are independently recognized as prognostic markers, the association between systemic inflammation (reflected by NLR) and acute neuronal injury (reflected by NSE) has not been sufficiently examined in large clinical populations. Elucidating this connection may enhance our understanding of the reciprocal interaction between peripheral immune responses and cerebral damage, potentially revealing a vicious cycle wherein inflammation exacerbates neuronal death and resultant damage‐associated molecular patterns further stimulate immune activation. The present study seeks to evaluate the association between NLR and serum NSE in a large cohort of AIS patients, thereby offering clinical insights into the interplay between circulating inflammatory cells and central neuronal injury after ischemic stroke.

## 2 Subjects and Methods

### 2.1 Ethics

This study was performed in line with the principles of the Declaration of Helsinki. Approval was granted by the Ethics Committee of the QingPu Branch of Zhongshan Hospital Affiliated to Fudan University, Shanghai, China (approval no: QY2023‐23). Written informed consent was obtained from all participants or their immediate family members in cases of impaired consciousness or dysarthria.

### 2.2 Design

A cross‐sectional analysis was performed using data from the Stroke Registry Database in our Hospital. Consecutive patients admitted with a diagnosis of AIS between January 1, 2015, and May 31, 2025, were considered for inclusion. This study aimed to investigate the relationship between NLR and neuronal injury in AIS by evaluating their circulating biomarkers. This study was reported in accordance with the STROBE statement. Because the study used routinely collected registry data, the reporting also considered the RECORD extension where applicable.

### 2.3 Study Subjects

Inclusion criteria: Patients were included if they met all of the following criteria, (1) diagnosis of AIS confirmed by clinical presentation and neuroimaging (CT or MRI) in accordance with current guidelines; (2) age 18 years or older; (3) time from symptom onset to hospital admission within 7 days (acute phase); and (4) availability of complete clinical and laboratory data necessary for analysis.

Exclusion Criteria: Individuals were excluded from the study for any of the following reasons, (1) diagnosis of transient ischemic attack (TIA) or stroke mimics without confirmed cerebral infarction; (2) presence of hemorrhagic stroke or mixed‐type stroke; (3) pre‐existing neurological disorders that could confound outcome assessment (e.g., neurodegenerative diseases, intracranial tumors, or severe traumatic brain injury); (4) active systemic infection, chronic inflammatory conditions (e.g., rheumatoid arthritis, inflammatory bowel disease), use of immunosuppressive therapy, known active malignancy, or end‐stage renal disease at the time of enrollment; or (5) incomplete or missing essential laboratory or clinical data.

### 2.4 Treatment and Management

All patients received standard management for AIS following contemporary clinical guidelines, including antiplatelet therapy, anticoagulation where indicated, blood pressure control, and statin administration. Treatment strategies were tailored to individual patient characteristics and institutional protocols.

### 2.5 Data Collection and Laboratory Measurements

Demographic characteristics, medical history, and clinical data were collected at admission. Fasting venous blood samples for both NLR calculation and NSE measurement were obtained within 24 h of hospitalization. Neutrophil and lymphocyte counts were measured using an automated hematology analyzer. NLR was calculated as the ratio of absolute neutrophil count to absolute lymphocyte count. Given its markedly right‐skewed distribution, NLR was log_2_‐transformed for normalization to meet the assumptions of parametric statistical tests. Serum NSE levels were quantified using an electrochemiluminescence immunoassay (ECLIA, Roche Diagnostics). The intra‐ and inter‐assay coefficients of variation were < 5%. The established clinical upper limit of normal (ULN) in our laboratory is 16.3 μg/L, which was used to define elevated NSE. Blood samples with visible hemolysis were discarded at collection.

### 2.6 Statistical Analysis

The characteristics of study participants at baseline are presented by NSE level. Categorical variables presented as frequency counts and percentages were analyzed using chi‐square tests. Continuous variables were presented as means with standard deviations (SDs) for normal distribution data, which were analyzed by *t* tests, and they were expressed as medians with interquartile ranges (IQRs) for abnormal distribution data, which were analyzed by Mann–Whitney *U*‐tests. NLR level was right‐skewed distribution and log_2_‐transformed for analyses, and comparison of high NSE was grouped by tertiles of log_2_‐transformed NLR. Participants with missing data on either NSE or NLR were excluded from the analysis. No imputation was performed for missing values, as the proportion of missing data was relatively small.

The association between log_2_‐transformed NLR and high NSE was assessed by linear curve fitting analyses (generalized additive models), cumulative incidence function analyses, and multivariate logistic regression analysis. Baseline variables that showed a univariate relationship with high NSE and clinically relevant factors including TOAST classification (large‐artery atherosclerosis subtype) were selected into multivariate logistic regression model (*p* < 0.05). Both non‐adjusted and adjusted models were used, and stratified analyses and interaction testing were performed. The threshold level (turning point) of log_2_‐transformed NLR was determined by using the threshold effect analysis, and a two‐piecewise regression model was applied to validate the threshold effect of the log_2_‐transformed NLR levels on the high NSE using a smoothing function. Additionally, a receiver operating characteristic (ROC) curve was constructed to assess the discriminatory ability of log_2_(NLR) in predicting high NSE, and the optimal cut‐off value was determined using the Youden Index. Statistical analyses were performed using the Statistical Package for the Social Sciences Software (SPSS) (version 24.0, Chicago, IL, USA) and R (version 3.6.3). The statistical significance level was set at a two‐tailed *p* value of < 0.05.

## 3 Results

### 3.1 Baseline Characteristics

**Figure 1:**
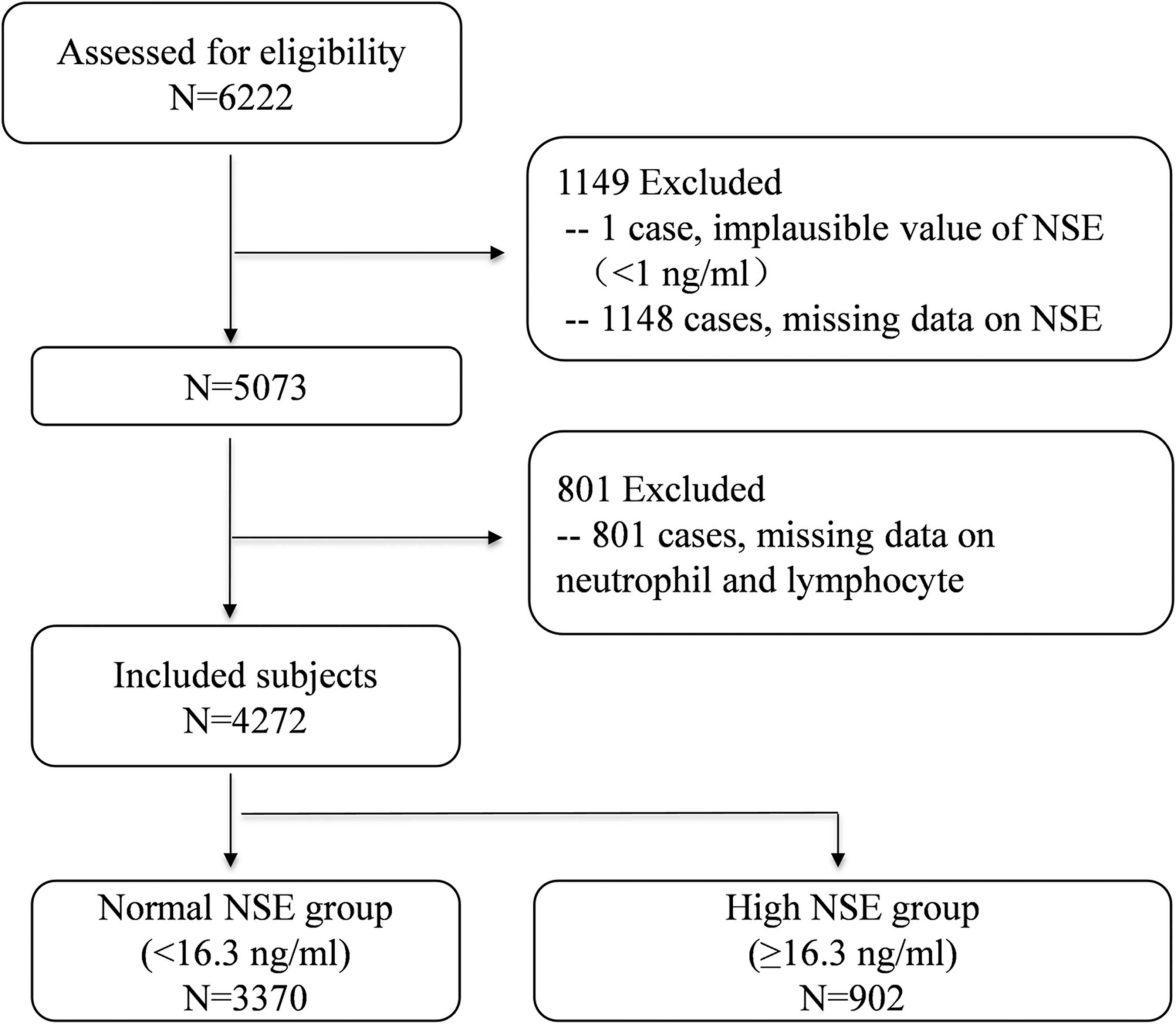
A flowchart of the study.

At the time of the final survey in May 2025, a total of 6222 consecutive AIS candidates were recruited for the study. Among these AIS candidates, one patient with an implausible value of NSE (< 1 μg/L) was excluded from the eligible candidates. Those with missing data related to NSE (*n* = 1148) and NLR (*n* = 801) were also excluded from the eligible candidates for the study. As a result, a total of 4272 AIS subjects were included for the final analyses. According to the clinical normal range of NSE, 4272 AIS subjects were categorized into normal and high NSE groups. A flowchart of the study is shown in Figure 1.

Among 4272 study AIS subjects, women accounted for 37.36% (*n* = 1596) and men for 62.64% (*n* = 2676). The age of the enrolled subjects ranged from 21 to 100 years (women, 34–100 years; men, 21–97 years) with a mean age of 69.50 ± 12.84 years (women, 73.69 ± 11.36 years; men, 67.00 ± 13.02 years). The disease duration before admission ranged from 0.5 to 50 h with a median and interquartile range of 11.0 (9.0–12.0) h. The NSE ranged from 4.14 to 217 μg/L, the neutrophil counts ranged from 0.52 to 31.53 × 10^12^/L, and the lymphocyte counts ranged from 0.14 to 12.73 × 10^12^/L. The NLR value (abnormal distribution) ranged from 0.27 to 83.56; correspondingly, log_2_‐transformed NLR value (normal distribution) ranged from −1.88 to 6.38 with a mean value of 1.63 ± 0.87 (Figure S1). The baseline characteristics of patients are shown in Table 1 and Table S1.

**Table 1:** Baseline characteristics of the participants by NSE levels (*n* = 4272).

Characteristics	Grouped by NSE level		*p*
	Normal NSE group (< 16.3 μg/L)	High NSE group (≥ 16.3 μg/L)	
Basic information			
*N*	3370	902	
Sex (men) (%)	2110 (62.61)	566 (62.75)	0.939
Age (years)	69.30 ± 12.45	70.25 ± 14.19	0.048
Disease duration (h)	10.88 ± 4.48	11.45 ± 5.36	0.001
TOAST‐LAA (%)	574 (17.03)	165 (18.29)	0.374
Medical history			
Hypertension (%)	2604 (77.27)	691 (76.61)	0.674
Diabetes (%)	1202 (35.67)	241 (26.72)	< 0.001
CHD (%)	227 (6.74)	91 (10.09)	< 0.001
AF (%)	2990 (88.72)	662 (73.39)	< 0.001
Blood indicators			
TC (mmol/L)	3.98 ± 1.02	3.94 ± 1.01	0.328
TG (mmol/L)	1.47 ± 0.97	1.32 ± 0.74	< 0.001
LDL‐C (mmol/L)	2.34 ± 0.85	2.36 ± 0.84	0.483
ALT (U/L)	21.00 (15.00–30.00)	25.00 (16.75–36.00)	< 0.001
Urea (mmol/L)	5.62 ± 2.86	6.21 ± 3.62	< 0.001
Creatinine (μmol/L)	63.00 (53.00–75.00)	63.00 (54.00–78.00)	0.052
UA (μmol/L)	277.18 ± 90.43	275.80 ± 104.37	0.172
FBG (mmol/L)	6.45 ± 2.49	6.70 ± 2.69	0.011
Glycosylated hemoglobin (%)	6.68 ± 1.69	6.55 ± 1.59	0.051
HCY (μmol/L)	12.20 (9.90–15.90)	13.60 (10.40–18.60)	< 0.001
Folic acid (ng/mL)	8.14 ± 4.71	8.42 ± 5.07	0.508
WBC (10^9^/L)	6.94 ± 2.44	7.58 ± 2.94	< 0.001
RBC (10^12^/L)	4.20 ± 0.61	4.25 ± 0.69	0.039
PLT (10^9^/L)	207.55 ± 70.21	223.02 ± 91.77	< 0.001
HGB (g/L)	128.50 ± 18.30	129.82 ± 20.23	0.059
Lymphocyte count (10^9^/L)	1.56 ± 0.73	1.44 ± 0.69	< 0.001
Neutrophil count (10^9^/L)	4.63 ± 2.16	5.32 ± 2.71	< 0.001
NLR	2.83 (2.03–4.05)	3.47 (2.29–5.37)	< 0.001
Log_2_ transformed NLR	1.56 ± 0.82	1.88 ± 1.00	< 0.001
NSE (μg/L)	12.04 ± 2.16	22.80 ± 13.79	< 0.001
Thyroxine (μg/dL)	91.79 ± 19.76	94.84 ± 19.92	< 0.001
Tumor index			
AFP (ng/mL)	2.14 (1.55–3.01)	2.20 (1.62–3.25)	0.024
CEA (ng/mL)	2.40 (1.60–3.50)	2.70 (1.70–3.80)	0.006
Medication use on admission			
Antiplatelet drugs (%)	2595 (77.00)	634 (70.29)	< 0.001
Anticoagulant drugs (%)	667 (19.79)	207 (22.95)	0.037
Lipid‐lowering drugs (%)	2972 (88.19)	785 (87.03)	0.342
Antihypertensive drugs (%)	2276 (67.54)	579 (64.19)	0.058
Antidiabetic drugs (%)	2326 (69.02)	625 (69.29)	0.876

*Note:* High NSE was defined as serum NSE ≥ 16.3 μg/L, the upper limit of normal in our laboratory.

Abbreviations: AF, atrial fibrillation; AFP, alpha fetoprotein; ALT, alanine aminotransferase; CEA, carcinoembryonic antigen; CHD, coronary heart disease; FBG, fasting blood glucose; HCY, homocysteine; HGB, hemoglobin; LAA, large‐artery atherosclerosis; LDL‐C, low‐density lipoprotein cholesterol; NLR, neutrophil‐to‐lymphocyte ratio; NSE, neuron‐specific enolase; PLT, platelet; RBC, red blood cell; TC, total cholesterol; TG, triglyceride; TOAST, Trial of Org 10172 in Acute Stroke Treatment; UA, uric acid; WBC, white blood cell.

### 3.2 Differential Expression Levels of NSE and Prevalence of High NSE Grouped by Tertiles of log_2_‐Transformed NLR

**Figure 2:**
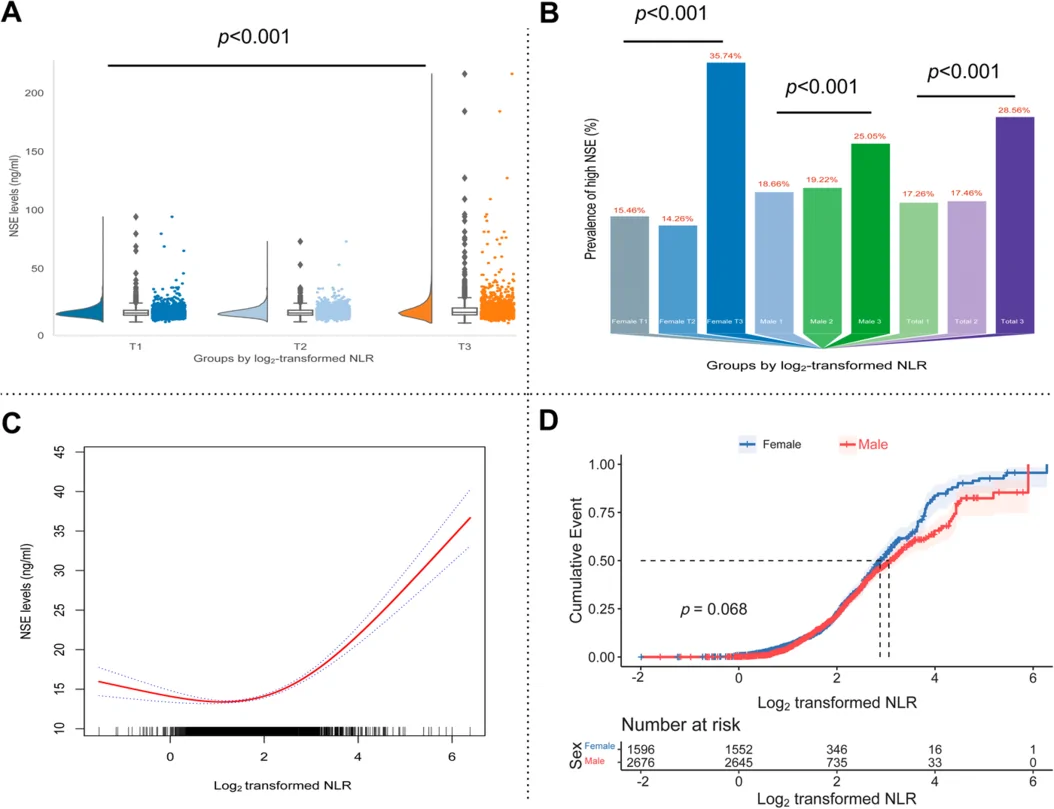
Differential expression levels of NSE and prevalence of high NSE according to log_2_‐transformed NLR. (A) Serum NSE levels showed significant differences among low (T1), middle (T2), and high (T3) log_2_‐transformed NLR groups (*p* < 0.001). (B) Prevalence of high NSE increased with the increasing of log_2_‐transformed NLR levels (*p* < 0.001). (C) Smoothed plots showed linear relationships between log_2_‐transformed NLR and NSE (*p* < 0.001). (D) Cumulative incidence function analyses observed increasing cumulative event rates of high NSE (*p* < 0.001).

In all included AIS patients, the mean serum NSE levels were 13.61 ± 5.38 μg/L, 13.42 ± 4.23 μg/L, and 15.89 ± 11.76 μg/L in the low (T1, *n* = 1414), middle (T2, *n* = 1426), and high (T3, *n* = 1432) log_2_‐transformed NLR tertiles for all patients, respectively, and there was a significant difference among the three groups (*p* < 0.001) (Figure 2A). Prevalence of high NSE were 17.26%, 17.46%, and 28.56% in the low (T1), middle (T2), and high (T3) log_2_‐transformed NLR tertiles for all patients (*p* < 0.001) (Figure 2B), respectively. Subgroup analysis by sex also observed a significant difference both in NSE levels and prevalence of high NSE (both *p* < 0.001) (Table S2). These results indicated that both serum NSE levels and prevalence of high NSE were increased with the increasing of log_2_‐transformed NLR levels.

### 3.3 Linear Curve Fitting of the Relationship Between log_2_‐Transformed NLR and NSE and High NSE, Respectively

Smoothed plots revealed linear relationships between log_2_‐transformed NLR and NSE after adjusting for sex, age, disease duration, CHD, TG, ALT, urea, creatinine, FBG, HCY, RBC, PLT, thyroxine, antiplatelet drugs, and anticoagulant drugs (*p* < 0.001) (Figure 2C). Cumulative incidence function analyses showed that cumulative event rates of high NSE were increasing according to log_2_‐transformed NLR levels, and both women and men observed the same increasing cumulative event (both *p* < 0.001) (Figure 2D).

### 3.4 Multiple Logistic Regression Analyses of the Relationship Between log_2_‐Transformed NLR and High NSE

**Figure 3:**
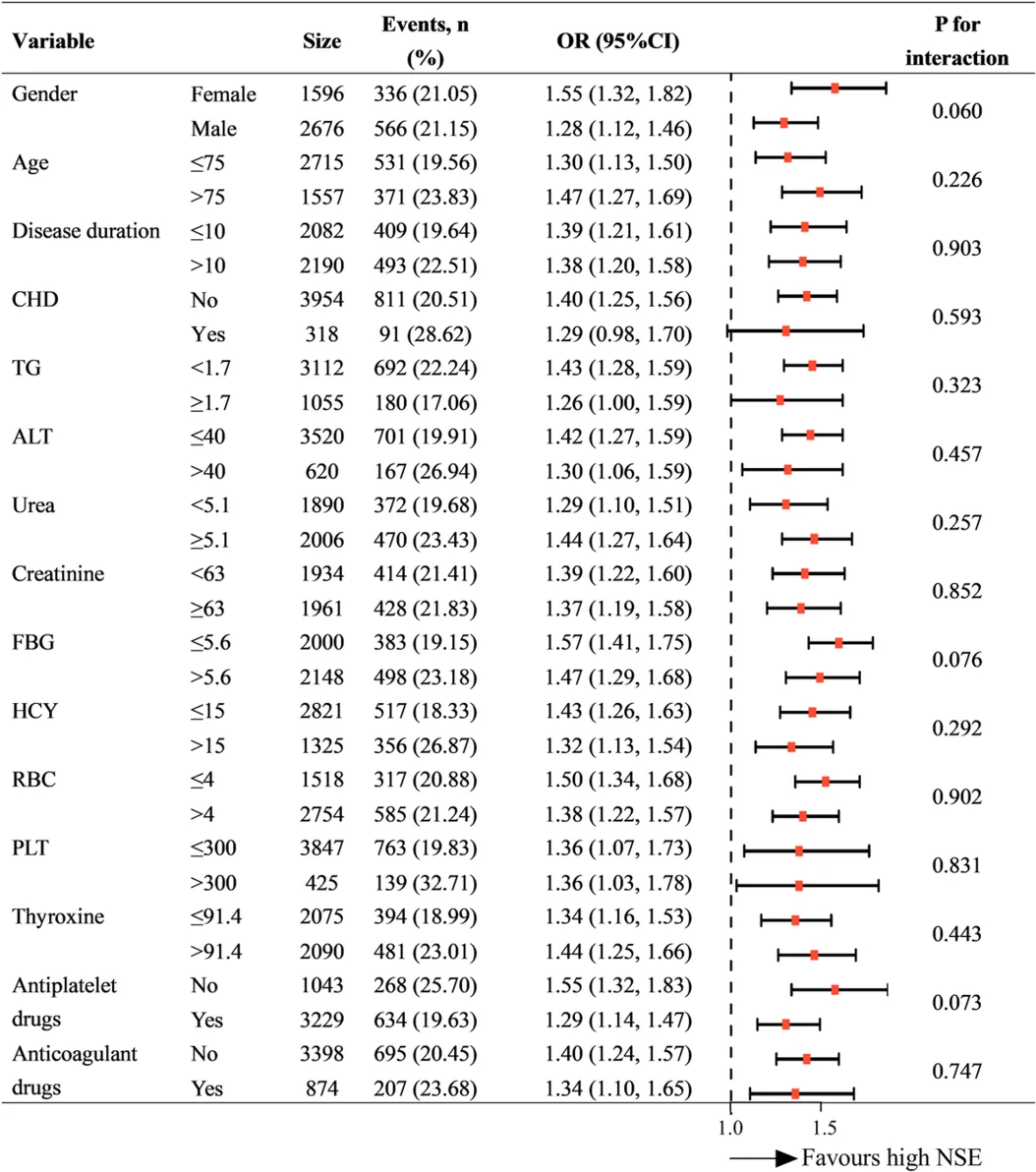
Forest plot of hierarchical analysis on the relationship. Hierarchical analysis of the relationship of log_2_‐transformed NLR and high NSE. Each stratification was adjusted for all the factors (sex, age, disease duration, CHD, TG, ALT, urea, creatinine, FBG, HCY, RBC, PLT, thyroxine, antiplatelet drugs, and anticoagulant drugs) except the stratification factor itself.

In multiple logistic regression analyses of the relationship between log_2_‐transformed NLR and high NSE, baseline variables that were considered relevant to NSE and log_2_‐transformed NLR by difference analysis were selected for univariate analysis. Sex, age, and those factors with *p* < 0.05 in univariate analysis (Table S3) were included in the multivariate logistic regression model. As a result, sex, age, disease duration, CHD, TG, ALT, urea, creatinine, FBG, HCY, RBC, PLT, thyroxine, antiplatelet drugs, and anticoagulant drugs were identified as confounding factors related to high NSE. In multiple logistic regression analysis, the OR (95%) for the relationship between log_2_‐transformed NLR and high NSE were 1.50 (1.38–1.63, *p* < 0.001) and 1.38 (1.25–1.53, *p* < 0.001) in all patients before and after adjusting for sex, age, disease duration, CHD, TG, ALT, urea, creatinine, FBG, HCY, RBC, PLT, thyroxine, antiplatelet drugs, and anticoagulant drugs. In the present study, a one‐unit increment in the log_2_‐transformed NLR corresponds to a twofold increase in the original NLR value. This implies that, when adjusting for potential confounders, a twofold elevation in NLR is associated with a 1.38‐fold risk of high NSE. After adjusted confounder factors, the OR (95%) for the relationship between log_2_‐transformed NLR and high NSE were 1.60 (1.35–1.89, *p* < 0.001) and 1.27 (1.11–1.44, *p* < 0.001) in women and men, respectively, and the OR (95%) showed a graded increase according to the log_2_‐transformed NLR tertiles by trend analysis (*p* < 0.001) (Table 2), which showed a statistical significance. Both sensitivity analysis and hierarchical analysis, according to sex, age, disease duration, CHD, TG, ALT, urea, creatinine, FBG, HCY, RBC, PLT, thyroxine, antiplatelet drugs, and anticoagulant drugs, also showed that the association between log_2_‐transformed NLR and high NSE was statistically significant (Figure 3, Tables S4 and S5).

**Table 2:** Multivariate logistic regression for effects of log_2_‐transformed NLR on high NSE.

Patients, *N*	Model 1 (unadjusted)			Model 2 (adjusted)	
	*N*	Incidence, *n* (%)	OR (95% CI), *p*	*N*	OR (95% CI), *p*
Log_2_‐transformed NLR (continuous) mmol/L	4272	902 (21.11%)	1.50 (1.38, 1.63), < 0.001	3550	1.38 (1.25, 1.53), < 0.001
Log_2_‐transformed NLR (categorical)					
Female	1956	336 (21.05%)	1.84 (1.60, 2.10), < 0.001	1327	1.60 (1.35, 1.89), < 0.001
Male	2676	566 (21.15%)	1.31 (1.18, 1.46), < 0.001	2223	1.27 (1.11, 1.44), < 0.001
Log_2_‐transformed NLR (categorical)					
T 1 (−1.88 to 1.22) mmol/L	1414	244 (17.26%)	1.0 (reference)	1181	1.0 (reference)
T 2 (1.23–1.89) mmol/L	1426	249 (17.46%)	1.01 (0.84, 1.23), 0.885	1183	1.00 (0.80, 1.24), 0.976
T 3 (1.90–6.38) mmol/L	1432	409 (28.56%)	1.92 (1.60, 2.29), < 0.001	1186	1.53 (1.23, 1.89), < 0.001
*p* for trend	4272		1.58 (1.40, 1.78), < 0.001	3550	1.34 (1.17, 1.55), < 0.001

*Note:* High NSE was defined as serum NSE ≥ 16.3 μg/L, the upper limit of normal in our laboratory. Model 1: unadjusted; Model 2: adjusted for sex, age, disease duration, CHD, TG, ALT, urea, creatinine, FBG, HCY, RBC, PLT, thyroxine, antiplatelet drugs, and anticoagulant drugs.

### 3.5 Threshold Effect Analysis to Determine Kink (*K*) Value for Effects of log_2_‐Transformed NLR on High NSE

**Figure 4:**
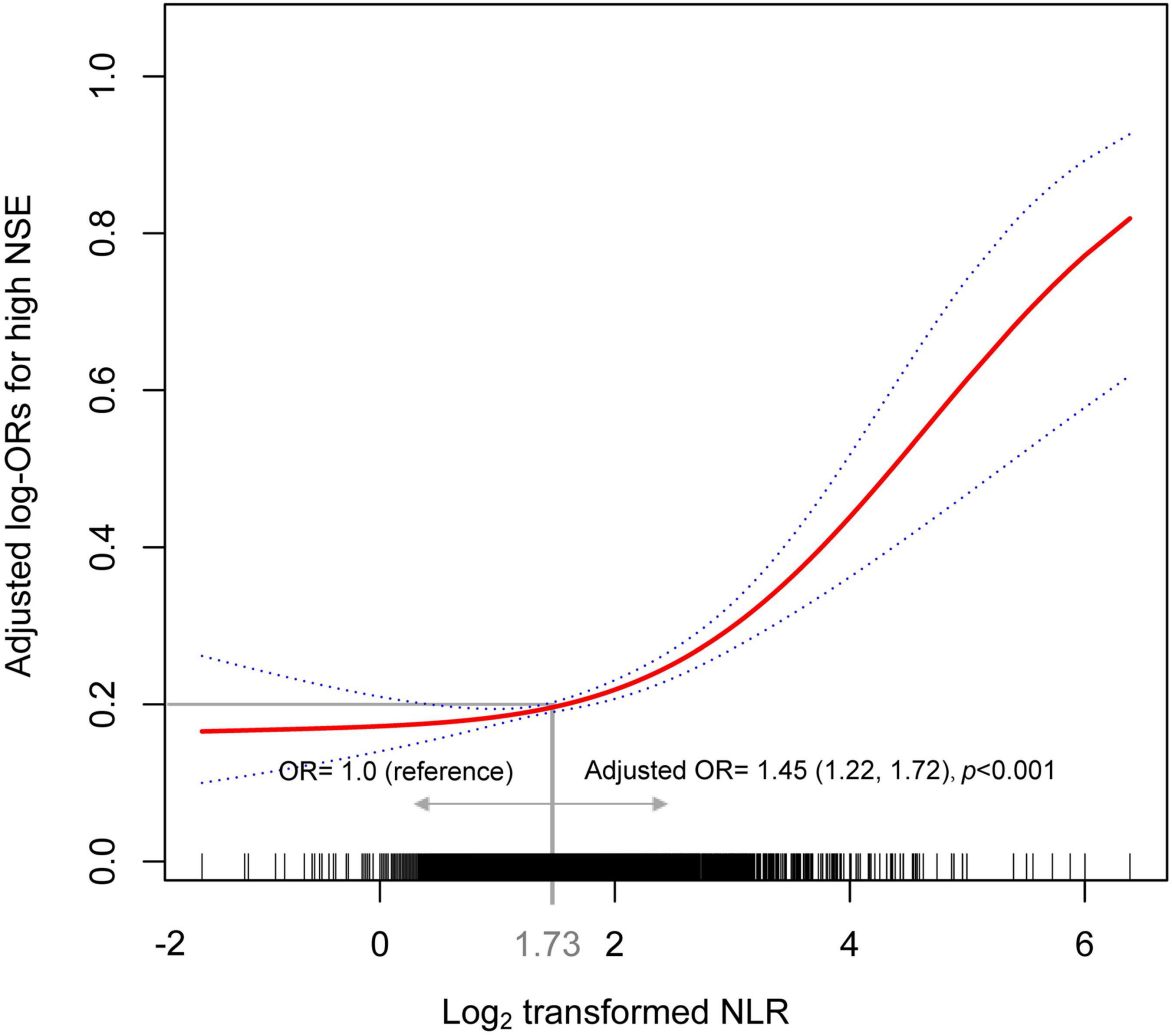
Threshold effect analysis on risk of high NSE. Threshold effect analysis detected that 1.73 was the *K* value for two‐segment effects of log_2_‐transformed NLR on high NSE, and the probability of high NSE significantly increased when log_2_‐transformed NLR > 1.73 (*p* < 0.001).

Threshold effect analysis detected that 1.73 was the *K* value for two‐segment effects of log_2_‐transformed NLR on high NSE, and the probability of high NSE significantly increased with the increment of log_2_‐transformed NLR levels in K2 (> 1.73) compared to K1 (< 1.73) segment patients, which showed a significantly difference (OR = 1.75 [1.29–2.39], *p* < 0.001) (Table S6). To validate the potential risk of the *K* value (log_2_‐transformed NLR = 1.73), prevalence and risk of high NSE were compared between the K1 and K2 segment patients grouped by the *K* value, and prevalence and risk of high NSE were 17.05% and 26.92% in the K1 and K2 patients (*p* < 0.001). After adjusting for confounder factors, the OR (95%) for the relationship between log2‐transformed NLR and high NSE was 1.0 (reference) and 1.45 (1.22–1.72, *p* < 0.001) in K1 and K2 segment patients, respectively (Figure 4). These analyses also showed that the risk of high NSE was increased with the increment of log_2_‐transformed NLR levels (Table S7).

ROC analysis for the predictive value of log_2_(NLR) for high NSE yielded an area under the curve (AUC) of 0.62 (95% CI: 0.60–0.64). The optimal cutoff value determined by the Youden Index was log_2_(NLR) = 1.73 (corresponding to an NLR of approximately 3.3), with a sensitivity of 53% and a specificity of 68% (Figure S2).

## 4 Discussion

The present cross‐sectional study, comprising a large cohort of 4272 AIS patients, reveals a significant and independent association between the NLR and NSE levels after adjusting for multiple clinical confounders. These findings suggest a relevant link between systemic inflammation and the extent of central neuronal damage in AIS, providing epidemiological support for the interplay between peripheral immune responses and cerebral injury processes.

These observations align with the growing recognition of the “dual‐hit” pathological mechanism in ischemic stroke, where initial ischemic insult is rapidly compounded by an inflammatory response. Neutrophils, as key mediators of innate immunity, infiltrate the brain parenchyma within hours after stroke onset, releasing proteolytic enzymes, reactive oxygen species, and pro‐inflammatory cytokines such as IL‐1β and TNF‐α [8, 9, 10]. These substances contribute to blood–brain barrier disruption, amplify local inflammation, and exacerbate ischemic damage [11, 12]. Conversely, lymphocytes, particularly regulatory T cells and Th2 subsets, exhibit neuroprotective properties through the modulation of inflammatory cascades and promotion of tissue repair mechanisms [13, 14]. The NLR encapsulates this dynamic balance—elevations reflect a predominance of pro‐inflammatory neutrophils relative to anti‐inflammatory lymphocytes, indicating a systemic inflammatory state that may be associated with worse cerebral outcomes [15, 16]. Our data reinforce the association of NLR with stroke severity and extend its relevance to reflect the degree of acute neuronal injury, as quantified by NSE. Our findings are consistent with prior smaller studies that reported correlations between inflammatory ratios (like NLR or platelet‐to‐lymphocyte ratio) and markers of brain injury, such as S100B or NSE [17, 18]. However, our study substantially expands this evidence by demonstrating this association in a much larger, well‐characterized cohort with comprehensive adjustment for confounders.

The strong correlation between NLR and NSE also hints at a potential bidirectional relationship, although causality cannot be inferred from our cross‐sectional design. On one hand, systemic inflammation may intensify neuronal damage through leukocyte activation, endothelial dysfunction, and cytokine‐mediated toxicity [19]. On the other hand, products of neuronal injury are known to enter the circulation and activate peripheral immune cells, thereby further amplifying inflammatory responses [14]. Molecules like HMGB1, released from damaged neurons, can engage Toll‐like receptors on immune cells and stimulate the production of neutrophils and monocytes, creating a self‐sustaining cycle of inflammation and injury [20, 21, 22]. This vicious feedback loop may help explain why higher NLR values correspond to greater central nervous system damage, as indicated by elevated NSE [23].

Notably, the log_2_ transformation of NLR was necessary to achieve normal distribution for our regression models. The inflection point identified at log_2_(NLR) = 1.73 (NLR ≈ 3.3) and confirmed by ROC analysis suggests a threshold beyond which the probability of high NSE rises markedly. However, the modest AUC of 0.62 indicates that NLR alone has limited discriminatory power for identifying patients with high NSE. This finding underscores the distinction between a statistical association and clinical utility, highlighting the role of NLR as one component within a multifactorial pathological process rather than a standalone diagnostic or predictive tool. This aligns with prior observational studies in AIS that have cautioned against overinterpreting similar biomarker associations [24]. The identified threshold may serve as a candidate for future research into multi‐marker risk stratification protocols, but external validation is warranted before any clinical application. When contextualized within existing literature, our findings corroborate and extend previous work. For instance, NLR has been associated with functional outcome, whereas NSE levels are predictive of long‐term neurological recovery [25]. Our analysis provides population‐level evidence linking peripheral inflammatory status with a direct biomarker of neuronal injury. Moreover, the consistency of our results across subgroup and sensitivity analyses strengthens the validity of the conclusions.

## 5 Limitations

Several limitations of this study merit consideration. First, the cross‐sectional design precludes causal inference regarding the directionality of the observed association. We cannot determine whether systemic inflammation (reflected by NLR) contributes to neuronal injury or if neuronal injury (reflected by NSE) amplifies the inflammatory response. Furthermore, the use of a single time‐point measurement limits our understanding of the temporal dynamics between these two biomarkers. Second, despite extensive adjustment for confounders, residual confounding from unmeasured variables such as pre‐existing subclinical inflammation, infarct volume, or admission NIHSS scores (which were not consistently available in our registry) cannot be entirely excluded. Third, while we excluded patients with active infections and discarded visibly hemolyzed samples, unmeasured confounders such as subclinical infections or subclinical hemolysis could potentially influence NLR and NSE levels, respectively. Fourth, all participants were recruited from a single center in China, which may affect the generalizability of the findings to other populations or healthcare settings. Fifth, the lack of a healthy control group or a non‐stroke hospitalized patient group limits our ability to determine the specificity of the observed NLR‐NSE relationship to AIS pathophysiology versus a general acute illness response. Finally, the modest discriminatory performance of NLR reinforces that its clinical implications remain speculative and warrant further interventional investigation, likely as part of a multi‐biomarker panel.

## 6 Conclusion

In conclusion, this study demonstrates a significant association between elevated NLR and high serum NSE levels in patients with AIS, supporting an association between systemic inflammation and neuronal injury. Given the cross‐sectional design, these findings should be interpreted as hypothesis‐generating. Future research should focus on elucidating the temporal and mechanistic pathways linking peripheral immune activation and cerebral damage through longitudinal studies, as well as exploring the utility of these biomarkers in guiding immunomodulatory therapies for ischemic stroke.

## Author Contributions

**Ning Rong:** data curation, validation. **Hongyan Li:** data curation, writing – original draft. **Siyu Qian:** formal analysis, writing – original draft. **Yiting Ya:** writing – review and editing. **Hongfu Tian:** data curation. **Ningning Wang:** supervision, data curation. **Yuehua Yu:** formal analysis. **Desheng Zhu:** supervision, writing – review and editing, funding acquisition. **Minyuan Liu:** supervision, project administration, writing – review and editing, funding acquisition. All authors have read and approved the final manuscript.

## Ethics Statement

This study was performed in line with the principles of the Declaration of Helsinki. Approval was granted by the Ethics Committee of the QingPu Branch of Zhongshan Hospital Affiliated to Fudan University, Shanghai, China (approval no: QY2023‐23).

## Consent

Written informed consent was obtained from all participants or their immediate family members in cases of impaired consciousness or dysarthria.

## Conflicts of Interest

The authors declare no conflicts of interest.

## Supporting information

Supporting File

Supporting File

Supporting File

## Data Availability

The data sets generated during the current study are not publicly available due to institutional and patient privacy restrictions, but are available from the corresponding author (Min‐Yuan Liu) upon reasonable request.
