# Supplementary material for: Systemic Inflammation and Central Neuronal Damage: The Relationship Between Neutrophil‐to‐Lymphocyte Ratio and Neuron‐Specific Enolase in Acute Ischemic Stroke
Source: Immun Inflamm Dis. 2026 Jun 4;14(6):e70456. doi: 10.1002/iid3.70456 (PMC13581072; doi:10.1002/iid3.70456)
Supplement: Supplementary file 1 [file IID3-14-e70456-s001.pdf]

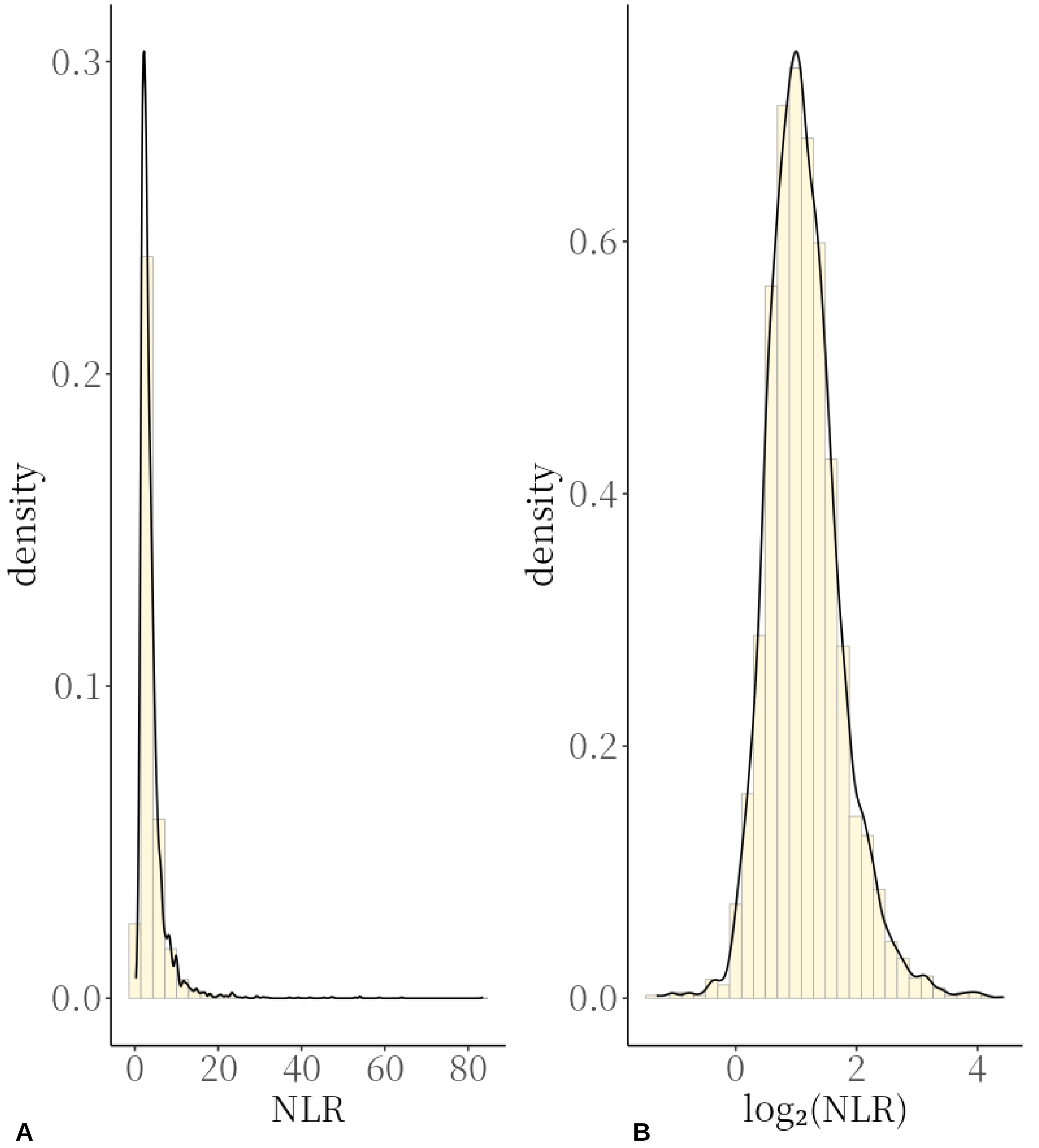

0.3
0.2
0.1
0.0
density
0
20
40
60
80
NLR
A
0.6
0.4
0.2
0.0
density
0
2
4
log₂(NLR)
B
