# Supplementary material for: Systemic Inflammation and Central Neuronal Damage: The Relationship Between Neutrophil‐to‐Lymphocyte Ratio and Neuron‐Specific Enolase in Acute Ischemic Stroke
Source: Immun Inflamm Dis. 2026 Jun 4;14(6):e70456. doi: 10.1002/iid3.70456 (PMC13581072; doi:10.1002/iid3.70456)
Supplement: Supplementary file 2 [file IID3-14-e70456-s003.pdf]

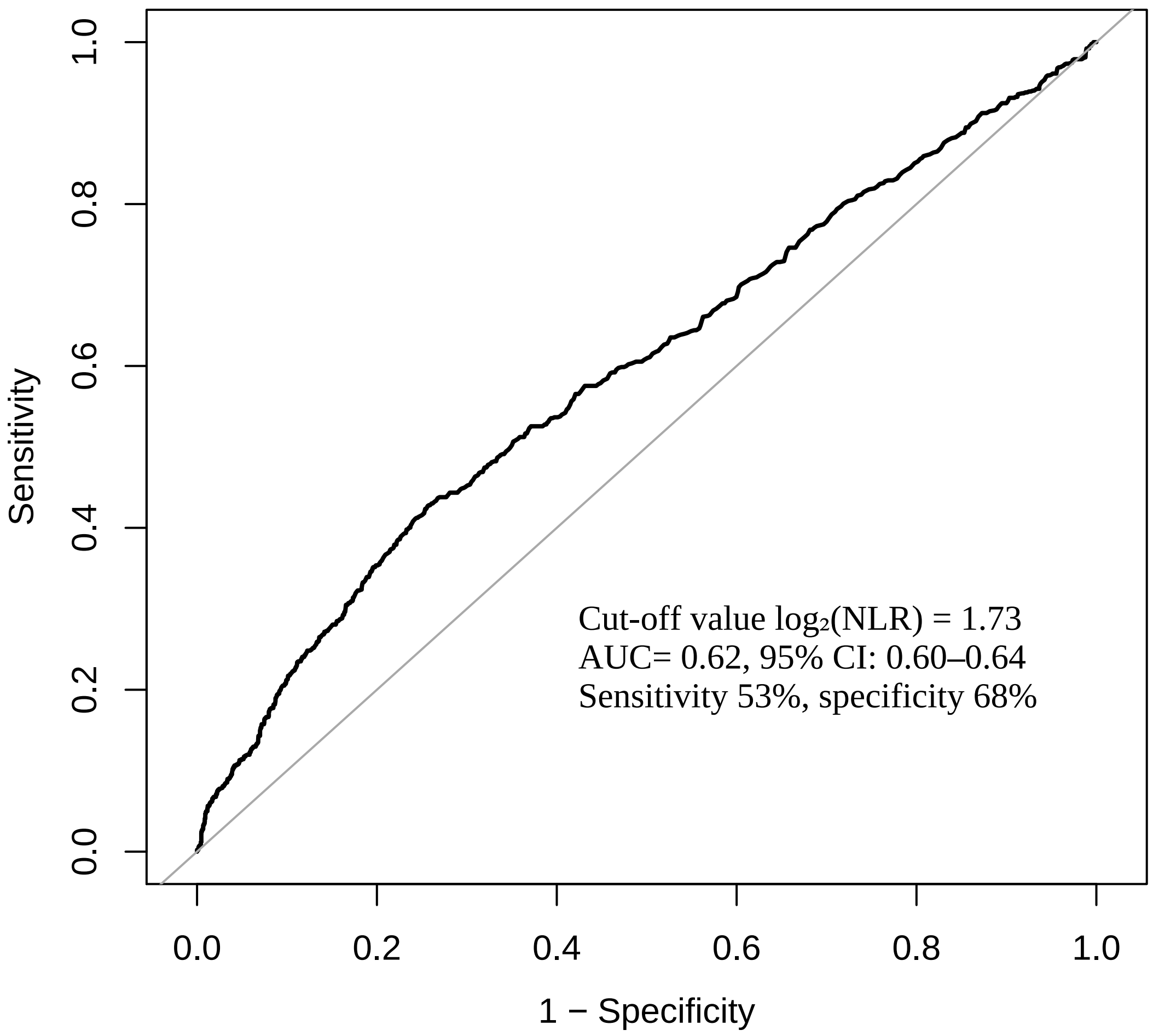

Cut-off value log₂(NLR) = 1.73
AUC= 0.62, 95% CI: 0.60–0.64
Sensitivity 53%, specificity 68%
Sensitivity
1 − Specificity
0.0
0.2
0.4
0.6
0.8
1.0
