# Supplementary material for: Systemic Inflammation and Central Neuronal Damage: The Relationship Between Neutrophil‐to‐Lymphocyte Ratio and Neuron‐Specific Enolase in Acute Ischemic Stroke
Source: Immun Inflamm Dis. 2026 Jun 4;14(6):e70456. doi: 10.1002/iid3.70456 (PMC13581072; doi:10.1002/iid3.70456)
Supplement: Supplementary file 3 [file IID3-14-e70456-s002.docx]

**Supplement Tables**

[Supplement Table 1. Baseline characteristics of participants by tertiles of log_2_-transformed NLR	2](#_Toc207750483)

[Supplement Table 2. NSE levels and prevalence of high NSE by log_2_-transformed NLR and gender	4](#_Toc207750484)

[Supplement Table 3. Univariate analysis of High NSE	5](#_Toc207750485)

[Supplement Table 4. Sensitivity analysis on relationship of log_2_-transformed NLR and high NSE	6](#_Toc207750486)

[Supplement Table 5. Hierarchical analysis on relationship of log_2_-transformed NLR and high NSE	7](#_Toc207750487)

[Supplement Table 6. Threshold effect analysis to determine K value for effects of log_2_-transformed NLR on high NSE	10](#_Toc207750488)

[Supplement Table 7. Two-piecewise regression analyses of log_2_-transformed NLR on high NSE by K values	11](#_Toc207750489)

# Supplement Table 1. Baseline characteristics of participants by tertiles of log_2_-transformed NLR

| **Characteristics** | **Log_2_ transformed NLR** | | | ***P*-value** |
| --- | --- | --- | --- | --- |
|  | **T1**  **Low group** | **T2**  **Middle group** | **T3**  **High group** |  |
| **Basic information** |  |  |  |  |
| N | 1414 | 1426 | 1432 |  |
| Sex (men) (%) | 793 (56.08) | 921 (64.59) | 962 (67.18) | <0.001 |
| Age (years) | 67.23±13.00 | 68.78±12.47 | 72.47±12.49 | <0.001 |
| Disease duration(hours) | 10.34±3.99 | 10.80±3.78 | 11.85±5.86 | <0.001 |
| TOAST-LAA (%) | 195 (13.79) | 246 (17.25) | 298 (20.81) | <0.001 |
| **Medical history** |  |  |  |  |
| Hypertension (%) | 1051 (74.33) | 1125 (78.89) | 1119 (78.14) | 0.008 |
| Diabetes (%) | 493 (34.87) | 506 (35.48) | 444 (31.01) | 0.023 |
| CHD (%) | 104 (7.36) | 94 (6.59) | 120 (8.38) | 0.188 |
| AF (%) | 175 (12.38) | 157 (11.01) | 288 (20.11) | <0.001 |
| **Blood indicators** |  |  |  |  |
| TC (mmol/L) | 3.98±1.02 | 3.95±0.97 | 3.97±1.06 | 0.921 |
| TG (mmol/L) | 1.57±1.09 | 1.46±0.89 | 1.28±0.76 | <0.001 |
| LDL-C (mmol/L) | 2.32±0.83 | 2.35±0.82 | 2.36±0.89 | 0.372 |
| ALT(U/L) | 27.36±21.73 | 26.12±19.92 | 32.66±77.55 | 0.041 |
| Urea (mmol/L) | 5.29±2.81 | 5.43±2.10 | 6.50±3.82 | <0.001 |
| Creatinine (μmol/L) | 66.50±49.18 | 68.49±36.05 | 78.53±82.74 | <0.001 |
| UA (μmol/L) | 286.62±91.93 | 281.88±88.80 | 262.54±98.03 | <0.001 |
| FBG (mmol/L) | 6.23±2.39 | 6.50±2.44 | 6.78±2.72 | <0.001 |
| Glycosylated hemoglobin (%) | 6.69±1.73 | 6.75±1.71 | 6.51±1.56 | <0.001 |
| HCY (μmol/L) | 13.83±9.91 | 14.64±9.87 | 16.69±11.59 | <0.001 |
| Folic acid (ng/mL) | 8.82±4.98 | 8.00±4.62 | 7.76±4.69 | <0.001 |
| WBC (10^9^/L) | 6.09±1.93 | 6.76±1.86 | 8.36±3.14 | <0.001 |
| RBC (10^12^/L) | 4.26±0.61 | 4.27±0.61 | 4.11±0.66 | <0.001 |
| PLT (10^9^/L) | 205.14±64.78 | 212.05±73.76 | 215.20±86.10 | 0.080 |
| HGB(g/L) | 130.77±17.53 | 130.26±17.81 | 125.33±20.23 | <0.001 |
| Lymphocyte count (10^9^/L) | 1.99±0.89 | 1.52±0.44 | 1.09±0.41 | <0.001 |
| Neutrophil count (10^9^/L) | 3.36±1.10 | 4.47±1.32 | 6.47±2.83 | <0.001 |
| NLR | 1.75±0.39 | 2.95±0.40 | 6.86±5.71 | <0.001 |
| Log_2_ transformed NLR | 0.76±0.40 | 1.55±0.19 | 2.57±0.66 | <0.001 |
| NSE (μg/L) | 13.61±5.38 | 13.42 ± 4.23 | 15.89±11.76 | <0.001 |
| Thyroxine (μg/dL) | 91.42±20.84 | 93.06±17.97 | 92.81±20.56 | 0.004 |
| Tumor index |  |  |  |  |
| AFP (ng/ml) | 7.07±168.19 | 2.51±2.00 | 2.41±1.43 | 0.025 |
| CEA (ng/ml) | 2.73±1.95 | 2.88±3.17 | 5.49±66.66 | <0.001 |
| **Medication use on admission** |  |  |  |  |
| Antiplatelet drugs (%) | 1111 (78.57) | 1111 (77.91) | 1007 (70.32) | <0.001 |
| Anticoagulant drugs (%) | 265 (18.74) | 286 (20.06) | 323 (22.56) | 0.037 |
| Lipid lowering drugs (%) | 1249 (88.33) | 1281 (89.83) | 1227 (85.68) | 0.003 |
| Antihypertensive drugs (%) | 966 (68.32) | 998 (69.99) | 891 (62.22) | <0.001 |
| Antidiabetic drugs (%) | 475 (33.59) | 450 (31.56) | 396 (27.65) | 0.002 |

**Abbreviations:** TOAST: Trial of Org 10172 in Acute Stroke Treatment, LAA: Large-artery atherosclerosis, CHD: Coronary heart disease, AF: Atrial fibrillation, TC: Total cholesterol, TG, Triglyceride, LDL-C: Low density lipoprotein cholesterol, ALT: Alanine aminotransferase, UA: Uric acid, FBG: Fasting blood glucose, HCY: Homocysteine, WBC: White blood cell, RBC: Red blood cell, PLT: Platelet, HGB: Hemoglobin, NLR: Neutrophil-to-lymphocyte ratio, NSE: Neuron-specific enolase, AFP: Alpha fetoprotein, CEA: Carcinoembryonic antigen.

# Supplement Table 2. NSE levels and prevalence of high NSE by log_2_-transformed NLR and gender

| **Log_2_-transformed NLR** | **Tertile 1** | **Tertile 2** | **Tertile 3** | ***P* value** |
| --- | --- | --- | --- | --- |
| **Femal**e |  |  |  |  |
| Number of patients | 621 | 505 | 470 |  |
| Log_2_-transformed NLR, mean (mmol/L) | 0.72±0.41 | 1.54±0.20 | 2.60±0.70 | <0.001 |
| NSE level, mean (μg/L) | 13.29±4.27 | 13.26±3.87 | 17.56±14.22 | <0.001 |
| Prevalence of high NSE (%) | 96 (15.46) | 72 (14.26) | 168 (35.74) | <0.001 |
| **Male** |  |  |  |  |
| Number of patients | 793 | 921 | 962 |  |
| Log_2_-transformed NLR, mean (mmol/L) | 0.79±0.38 | 1.56±0.19 | 2.56±0.64 | <0.001 |
| NSE level, mean (μg/L) | 13.87±6.11 | 13.51±4.41 | 15.07±10.25 | <0.001 |
| Prevalence of high NSE (%) | 148 (18.66) | 177 (19.22) | 241 (25.05) | <0.001 |
| **Total** |  |  |  |  |
| Number of patients | 1414 | 1426 | 1432 |  |
| Log_2_-transformed NLR, mean (mmol/L) | 0.76±0.40 | 1.55±0.19 | 2.57±0.66 | <0.001 |
| NSE level, mean (μg/L) | 13.61±5.38 | 13.42±4.23 | 15.89±11.76 | <0.001 |
| Prevalence of high NSE (%) | 244 (17.26) | 249 (17.46) | 409 (28.56) | <0.001 |

High NSE was defined as serum NSE ≥ 16.3 μg/L, the upper limit of normal in our laboratory.

# Supplement Table 3. Univariate analysis of High NSE

| Variable | Statistics | OR (95% CI) | *P-*value |
| --- | --- | --- | --- |
| Sex (%) |  |  |  |
| Women | 1596 (37.36) | 1.0 |  |
| Men | 2676 (62.64) | 1.01 (0.86, 1.17) | 0.939 |
| Age (years) | 69.50±12.84 | 1.01 (1.00, 1.01) | 0.048 |
| Disease duration(hours) | 11.00±4.69 | 1.02 (1.01, 1.04) | 0.001 |
| Diabetes (%) |  |  |  |
| No | 2829 (66.22) | 1.0 |  |
| Yes | 1443 (33.78) | 0.66 (0.56, 0.77) | <0.001 |
| TG (mmol/L) | 1.44±0.93 | 0.81 (0.73, 0.89) | <0.001 |
| ALT(U/L) | 28.71±47.96 | 1.00 (1.00, 1.01) | 0.002 |
| Urea (mmol/L) | 5.75±3.05 | 1.06 (1.03, 1.08) | <0.001 |
| Creatinine (μmol/L) | 71.24±59.82 | 1.00 (1.00, 1.00) | <0.001 |
| HGB(g/L) | 6.51±2.53 | 1.04 (1.01, 1.07) | 0.011 |
| HCY (μmol/L) | 15.05±10.55 | 1.02 (1.01, 1.02) | <0.001 |
| RBC (10^12^/L) | 4.21±0.63 | 1.13 (1.01, 1.27) | 0.039 |
| PLT (10^9^/L) | 210.82±75.53 | 1.00 (1.00, 1.00) | <0.001 |
| Thyroxine (μg/dL) | 92.43±19.83 | 1.01 (1.00, 1.01) | <0.001 |
| Antiplatelet drugs (%) |  |  |  |
| No | 1043 (24.41) | 1.0 |  |
| Yes | 3229 (75.59) | 0.71 (0.60, 0.83) | <0.001 |
| Anticoagulant drugs (%) |  |  |  |
| No | 3398 (79.54) | 1.0 |  |
| Yes | 874 (20.46) | 1.21 (1.01, 1.44) | 0.037 |

High NSE was defined as serum NSE ≥ 16.3 μg/L, the upper limit of normal in our laboratory.

# Supplement Table 4. Sensitivity analysis on relationship of log_2_-transformed NLR and high NSE

| **Variable** | **Model 1 (unadjusted)** | | **Model 2 (adjusted)** | | **Model 3 (adjusted)** | |
| --- | --- | --- | --- | --- | --- | --- |
|  | **N** | **OR (95% CI) *p*** | **N** | **OR (95% CI) *p*** | **N** | **OR (95% CI) *p*** |
| All patients | 4272 | 1.50 (1.38, 1.63) <0.001 | 4272 | 1.50 (1.38, 1.64) <0.001 | 3550 | 1.38 (1.25, 1.53) <0.001 |
| Hypertension | 3295 | 1.50 (1.36, 1.65) <0.001 | 3295 | 1.48 (1.34, 1.64) <0.001 | 2738 | 1.37 (1.21, 1.54) <0.001 |
| Diabetes | 1443 | 1.63 (1.38, 1.92) <0.001 | 1443 | 1.58 (1.34, 1.87) <0.001 | 1173 | 1.42 (1.16, 1.75) <0.001 |
| LDL-C >1.8 mmol/L | 3016 | 1.51 (1.37, 1.67) <0.001 | 3016 | 1.51 (1.36, 1.67) <0.001 | 2518 | 1.42 (1.26, 1.60) <0.001 |
| FBG >6.1 mmol/L | 1583 | 1.79 (1.57, 2.03) <0.001 | 1583 | 1.71 (1.49, 1.96) <0.001 | 1350 | 1.60 (1.36, 1.87) <0.001 |
| Lipid lowering drugs | 3757 | 1.54 (1.40, 1.69) <0.001 | 3757 | 1.53 (1.40, 1.68) <0.001 | 3130 | 1.40 (1.25, 1.56) <0.001 |

Model 1: unadjusted

Model 2: adjusted for sex and age.

Model 3: adjusted for sex, age, disease duration, CHD, TG, ALT, urea, creatinine, FBG, HCY, RBC, PLT, thyroxine, antiplatelet drugs, and anticoagulant drugs.

# Supplement Table 5. Hierarchical analysis on relationship of log_2_-transformed NLR and high NSE

| **Hierarchical factor** | | **Model 1 (unadjusted)** | | | | **Model 2 (adjusted)** | | |
| --- | --- | --- | --- | --- | --- | --- | --- | --- |
|  |  | **N** | **Events, n (%)** | **OR (95CI) *P*** | ***P* for interaction** | **N** | **OR (95CI) *P*** | ***P* for interaction** |
| Gender | Women | 1596 | 336 (21.05) | 1.84 (1.60, 2.10) <0.001 | 0.010 | 1327 | 1.55 (1.32, 1.82) <0.001 | 0.060 |
|  | Men | 2676 | 566 (21.15) | 1.31 (1.18, 1.46) <0.001 |  | 2223 | 1.28 (1.12, 1.46) <0.001 |  |
| Age | ≤75 | 2715 | 531 (19.56) | 1.29 (1.15, 1.46) <0.001 | 0.019 | 2238 | 1.30 (1.13, 1.50) <0.001 | 0.226 |
|  | >75 | 1557 | 371 (23.83) | 1.69 (1.50, 1.91) <0.001 |  | 1312 | 1.47 (1.27, 1.69) <0.001 |  |
| Disease duration | ≤10 | 2082 | 409 (19.64) | 1.53 (1.36, 1.72) <0.001 | 0.577 | 1719 | 1.39 (1.21, 1.61) <0.001 | 0.903 |
|  | >10 | 2190 | 493 (22.51) | 1.46 (1.30, 1.64) <0.001 |  | 1831 | 1.38 (1.20, 1.58) <0.001 |  |
| CHD | No | 3954 | 811 (20.51) | 1.53 (1.40, 1.67) <0.001 | 0.092 | 3288 | 1.40 (1.25, 1.56) <0.001 | 0.593 |
|  | Yes | 318 | 91 (28.62) | 1.24 (0.98, 1.55) 0.068 |  | 262 | 1.29 (0.98, 1.70) 0.071 |  |
| TG | <1.7 | 3112 | 692 (22.24) | 1.51 (1.37, 1.66) <0.001 | 0.276 | 2649 | 1.43 (1.28, 1.59) <0.001 | 0.323 |
|  | ≥1.7 | 1055 | 180 (17.06) | 1.34 (1.09, 1.63) 0.005 |  | 901 | 1.26 (1.00, 1.59) 0.053 |  |
| ALT | ≤40 | 3520 | 701 (19.91) | 1.55 (1.40, 1.70) <0.001 | 0.348 | 3008 | 1.42 (1.27, 1.59) <0.001 | 0.457 |
|  | >40 | 620 | 167 (26.94) | 1.40 (1.18, 1.67) <0.001 |  | 542 | 1.30 (1.06, 1.59) 0.011 |  |
| Urea | <5.1 | 1890 | 372 (19.68) | 1.38 (1.19, 1.59) <0.001 | 0.221 | 1751 | 1.29 (1.10, 1.51) 0.001 | 0.257 |
|  | ≥5.1 | 2006 | 470 (23.43) | 1.54 (1.38, 1.72) <0.001 |  | 1799 | 1.44 (1.27, 1.64) <0.001 |  |
| Creatinine | <63 | 1934 | 414 (21.41) | 1.48 (1.31, 1.67) <0.001 | 0.858 | 1765 | 1.39 (1.22, 1.60) <0.001 | 0.852 |
|  | ≥63 | 1961 | 428 (21.83) | 1.50 (1.33, 1.70) <0.001 |  | 1785 | 1.37 (1.19, 1.58) <0.001 |  |
| FBG | ≤5.6 | 2000 | 383 (19.15) | 1.31 (1.14, 1.50) <0.001 | 0.041 | 1721 | 1.57 (1.41, 1.75) <0.001 | 0.076 |
|  | >5.6 | 2148 | 498 (23.18) | 1.23 (1.05, 1.44) 0.011 |  | 1829 | 1.47 (1.29, 1.68) <0.001 |  |
| HCY | ≤15 | 2821 | 517 (18.33) | 1.48 (1.33, 1.66) <0.001 | 0.733 | 2421 | 1.43 (1.26, 1.63) <0.001 | 0.292 |
|  | >15 | 1325 | 356 (26.87) | 1.44 (1.26, 1.65) <0.001 |  | 1129 | 1.32 (1.13, 1.54) <0.001 |  |
| RBC | ≤4 | 1518 | 317 (20.88) | 1.51 (1.33, 1.71) <0.001 | 0.949 | 1276 | 1.50 (1.34, 1.68) <0.001 | 0.902 |
|  | >4 | 2754 | 585 (21.24) | 1.37 (1.17, 1.59) <0.001 |  | 2274 | 1.38 (1.22, 1.57) <0.001 |  |
| PLT | ≤300 | 3847 | 763 (19.83) | 1.50 (1.38, 1.64) <0.001 | 0.435 | 3178 | 1.36 (1.07, 1.73) 0.012 | 0.831 |
|  | >300 | 425 | 139 (32.71) | 1.40 (1.26, 1.56) <0.001 |  | 372 | 1.36 (1.03, 1.78) 0.029 |  |
| Thyroxine | ≤91.4 | 2075 | 394 (18.99) | 1.50 (1.33, 1.69) <0.001 | 0.961 | 1779 | 1.34 (1.16, 1.53) <0.001 | 0.443 |
|  | >91.4 | 2090 | 481 (23.01) | 1.50 (1.33, 1.69) <0.001 |  | 1771 | 1.44 (1.25, 1.66) <0.001 |  |
| Antiplatelet  drugs | No | 1043 | 268 (25.70) | 1.58 (1.38, 1.82) <0.001 | 0.178 | 865 | 1.55 (1.32, 1.83) <0.001 | 0.073 |
|  | Yes | 3229 | 634 (19.63) | 1.41 (1.27, 1.56) <0.001 |  | 2685 | 1.29 (1.14, 1.47) <0.001 |  |
| Anticoagulant  drugs | No | 3398 | 695 (20.45) | 1.49 (1.36, 1.64) <0.001 | 0.988 | 2809 | 1.40 (1.24, 1.57) <0.001 | 0.747 |
|  | Yes | 874 | 207 (23.68) | 1.49 (1.25, 1.79) <0.001 |  | 741 | 1.34 (1.10, 1.65) 0.004 |  |

Model 1: unadjusted

Model 2: each stratification adjusted for all the factors (sex, age, disease duration, CHD, TG, ALT, urea, creatinine, FBG, HCY, RBC, PLT, thyroxine, antiplatelet drugs, and anticoagulant drugs) except the stratification factor itself.

# Supplement Table 6. Threshold effect analysis to determine K value for effects of log_2_-transformed NLR on high NSE

| **Variable** | **Probability of high NSE** |
| --- | --- |
| **Model 1** |  |
| A straight-line effect | 1.38 (1.25, 1.53), *p*<0.001 |
| **Model 2** |  |
| Kink (K) value | 1.73 |
| < K1 segment effect | 1.00 (0.81, 1.22), *p=*0.979 |
| > K2 segment effect | 1.75 (1.48, 2.06), *p*<0.001 |
| The effect difference between K2 and K1 | 1.75 (1.29, 2.39), *p*<0.001 |
| Log-likelihood ratio tests | *p*<0.001 |

Model 1: Straight-line effect analysis.

Model 2: Threshold effect analysis.

Both model 1 and model 2 adjusted for sex, age, disease duration, CHD, TG, ALT, urea, creatinine, FBG, HCY, RBC, PLT, thyroxine, antiplatelet drugs, and anticoagulant drugs.

# Supplement Table 7. Two-piecewise regression analyses of log_2_-transformed NLR on high NSE by K values

| **Log_2_-transformed NLR (mmol/L)** | **Model 1(unadjusted)** | | **Model 2(adjusted)** | |
| --- | --- | --- | --- | --- |
|  | **N** | **OR (95%CI) *p*** | **N** | **OR (95%CI) *p*** |
| **Female** | 1596 |  | 1327 |  |
| < 1.73 | 1002 | 1.0 (reference) | 833 | 1.0 (reference) |
| ≥ 1.73 | 594 | 2.71 (2.12, 3.47) <0.001 | 494 | 1.96 (1.47, 2.63) <0.001 |
| **Male** | 2676 |  | 2223 |  |
| < 1.73 | 1509 | 1.0 (reference) | 1240 | 1.0 (reference) |
| ≥ 1.73 | 1167 | 1.41 (1.17, 1.70) <0.001 | 983 | 1.23 (0.99, 1.53) 0.067 |
| **Whole** | 4272 |  | 3550 |  |
| < 1.73 | 2511 | 1.0 (reference) | 2073 | 1.0 (reference) |
| ≥ 1.73 | 1761 | 1.80 (1.55, 2.08) <0.001 | 1477 | 1.45 (1.22, 1.72) <0.001 |

Model 1: unadjusted

Model 2: adjusted for adjusted for sex, age, disease duration, CHD, TG, ALT, urea, creatinine, FBG, HCY, RBC, PLT, thyroxine, antiplatelet drugs, and anticoagulant drugs.
